# Dental anomalies in cleft lip and/or palate children at age 10 - a retrospective review across three cleft centres: Part 1

**DOI:** 10.1038/s41415-023-5976-5

**Published:** 2023-06-23

**Authors:** Maryam Ezzeldin, Samantha Gee, Jacob Curtis, Victoria J. Clark, Jacqueline Smallridge, Mechelle Collard

**Affiliations:** 247976877746230946379 Specialist and Honorary Clinical Teacher in Paediatric Dentistry, University Dental Hospital and School, Cardiff, United Kingdom; 653950058805097653669 Specialty Registrar and Honorary Clinical Teacher in Paediatric Dentistry, University Dental Hospital and School, Cardiff, United Kingdom; 044583569523718993922 Post Certificate of Completion of Training in Orthodontics, Morriston Hospital, Swansea, United Kingdom; 058613338801689243125 Consultant in Paediatric Dentistry, Birmingham Children´s Hospital, United Kingdom; 008555186652816260154 Consultant in Paediatric Dentistry, CleftNetEast, Cambridge University Hospitals, United Kingdom; 151200456857680370237 Consultant in Paediatric Dentistry, Morriston Hospital, Swansea, UK; Consultant and Honorary Senior Lecturer in Paediatric Dentistry, University Dental Hospital and School, Cardiff, United Kingdom

## Abstract

**Introduction** Children with oral clefts often present with dental anomalies which can impact function, aesthetics and complicate the patient's dental treatment and needs. An understanding of potential anomalies, along with early recognition and planning, is thus essential for effective care.

**Aim** This paper is the first in a two-part three-centre series. This paper will assess the dental anomalies identified in 10-year-old patients attending three cleft centres in the UK.

**Method** Retrospective review was undertaken of the clinical notes of 10-year-old patients attending South Wales (SW), Cleft NET East (CNE) and West Midlands (WM) cleft units, for their ten-year audit record appointment in 2016/2017.

**Results** In total, 144 patients were reviewed (SW = 42; CNE = 52; WM = 50). Dental anomalies were recorded for 80.6% of patients (n = 116).

**Discussion** The review gives insight into the dental complexities of UK oral cleft patients. These patients require specialist paediatric dental input and intensive preventive regimes.

**Conclusion** Shared care between cleft team specialists and general dental practitioners is important when providing holistic care for cleft patients.

## Key points

- Patients with a cleft lip and/or palate (CLP) have been found to have a higher prevalence of dental anomalies when compared with the general population. These anomalies vary according to the CLP category and higher frequencies have been identified as the severity of the cleft increases.
- In view of the high prevalence of dental anomalies affecting CLP patients, specialist paediatric dental input in assessing and maintaining teeth and managing missing/malformed teeth with enhanced prevention is critical for CLP patients.
- It is important for dental practitioners working in primary care to understand these patients' dental needs and engage with cleft team specialists in providing shared holistic care for cleft patients.

## Introduction

Clefts of the lip and/or palate (CLP) are the most common congenital anomalies to affect the orofacial region, with a reported incidence of 15 in 10,000 live births within the UK.^1^ Cleft types vary according to severity and associated alveolar defects. The most common affects the palate only, accounting for approximately 44% of all clefts, as reported by the Cleft Registry and Audit Network.^1^ Clefts affecting both the lip and palate on one side only comprise 22% of all clefts, compared to those affecting both sides, which is the rarest and most severe type of cleft (10%). Isolated clefts of the lip represent 24% of all clefts, with some affecting the underlying alveolus.^1^

CLP can occur in isolation but often presents in combination with other congenital deformities and/or dental anomalies.^2^ Associated dental anomalies can have long-term impact on the patient's facial anatomy and self-esteem.^3^ Alleviating the functional and aesthetic consequences of CLP is particularly challenging.^4^ Associated medical conditions can impact cleft patients' dental risk and treatment, with conditions such as DiGeorge syndrome (22q11 deletion) for example having an increased risk of infective endocarditis, adding further complexity to these patients' holistic care.

Specialists in paediatric dentistry play an important role in the multidisciplinary care of this group of patients. Their involvement is key, not only because of the potential complexity of care due to associated medical conditions, but also the markedly increased incidence of dental anomalies in children with CLP compared to the general population.^5^^,^^6^ Shared care with general dental practitioners is paramount in the provision of positive early experiences, routine dental care, intensive preventive regimes and the overall holistic care to these patients.

This review looks to improve understanding of dental anomalies affecting CLP patients and the importance of effective shared care between cleft and primary dental teams in providing them with holistic dental care.

## Aim

The primary aim of this study is to assess the prevalence of dental anomalies currently affecting ten-year-old patients attending three cleft centres in the UK.

Through discussion we secondarily hope to:

- Describe the common dental anomalies found in CLP
- Illustrate the importance of early identification of dental anomalies
- Outline the value of shared care between specialist cleft and general dental service teams in the management of these patients.

These articles aim to provide the basis of knowledge required for understanding of potential complications in providing dental treatment for CLP where present with associated medical conditions. The prevalence of accompanying medical conditions and their relevance to the provision of dental care is to be further discussed in Part 2 of this two-part multi-centre series.^7^

##  Method

In this three-centre, cross-sectional study, retrospective data were collected from the clinical records and orthopantomogram radiographs where available of 10-year-old patients attending South Wales (SW), Cleft NET East (CNE) and West Midlands (WM) cleft units in 2016/2017. Patients in the three centres had been examined by calibrated paediatric dentistry specialists within the relevant cleft team. Much of the data were collected from audit data gathered nationally within the UK at age 10 years for all cleft lip and/or palate children.

Patients included were randomly selected from each cleft unit's databases using a random number generator. A minimum of 40 patient cases, who attended their 10-year cleft clinic and whose multidisciplinary summaries and clinical records could be accessed, were selected from each cleft unit.

Patient data gathered included: sex; type of cleft; dental anomalies; decayed, missing and filled teeth; medical conditions (to be discussed in Part 2);^7^ and whether they had undergone alveolar bone grafting. Data were collected using an Excel spreadsheet by four data collectors: one in WM, one in CNE and two in SW.

Dental anomalies were subcategorised under headings of: tooth agenesis; ectopic eruption/impaction; enamel hypomineralisation/hypoplasia; tooth shape/size anomalies; and supernumerary teeth.

Data were analysed using JASP (software version 0.11.1) and R (version 4.1.1, R Core Team, 2021). Fisher's exact test was performed for a 2x5 table, implemented in RStudio (www.r-studio.com), while chi-squared and Kruskal-Wallis H tests were conducted in JASP. A p-value less than 0.05 was considered significant.

## Results

In total, 144 patients were reviewed (SW = 42; CNE = 52; WM = 50) and of these, 42% (n = 61) were female and 58% (n = 83) were male. Dental anomalies were recorded for 80.6% of patients (n = 116), with distributions varying according to cleft type and associated alveolar defects (Table 1). Of patients with no reported dental anomalies, over four-fifths (n = 23; 82.1%) were cleft palate (CP) patients.

**Table 1:** Presence of dental anomalies by cleft type

Type of cleft	Total cases	Percentage of cases with dental anomalies (n)
Bilateral cleft lip and palate	17	94% (16)
Unilateral cleft lip and palate	53	96% (51)
Cleft lip and alveolus	17	100% (17)
Cleft lip	17	88% (15)
Cleft palate	40	43% (17)
**Totals**	**144**	

Dental anomalies and their prevalence by cleft type are summarised in , with the most common being enamel hypomineralisation/hypoplasia (HM/P) and tooth agenesis, affecting 45.8% (n = 66) and 41.6% (n = 60) of all patients, respectively. There were a greater number of dental anomalies recorded - a total of 206 - compared to the number of patients included in the review (n = 144). This is due to 65 patients (45.1%) presenting with more than one type of dental anomaly. Indeed, one patient reviewed presented with all five types of dental anomalies described. Chi-squared analyses indicated that tooth agenesis was most prevalent in bilateral CLP (BCLP) and unilateral CLP (UCLP) types, while ectopic eruption/impaction anomalies were more prevalent in BCLP types; both of which were significant findings. Enamel HM/P affected approximately 50% of all CLP types, but only 25% of CP-only patients. Supernumeraries were more prevalent in cleft lip and alveolus (CLA) and cleft lip (CL) patients, which was significant.

**Fig. 1:**
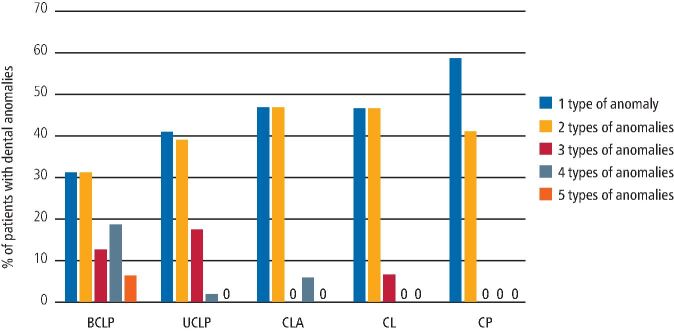
Presence of different types of dental anomalies in patients by cleft type

Certain cleft types were also shown to be more likely to present with a greater variety of different dental anomalies; with BCLP cleft type patients being more likely to develop three or more different dental anomalies (Fisher's exact test 2x5 table; p = 0.0199) (Fig. 1).

**Fig. 2:**
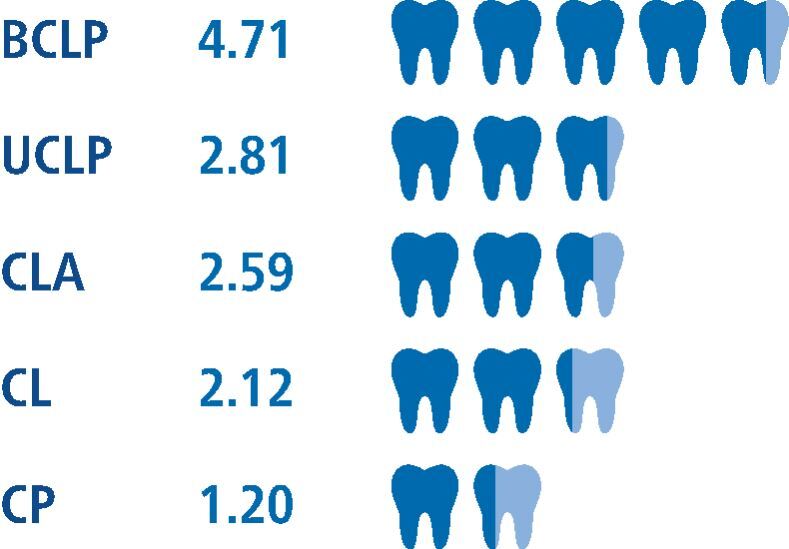
Mean number of teeth affected by dental anomalies within each cleft type

This is also reflected by the mean number of teeth affected by dental anomalies. There was variation between different cleft types, with BCLP patients presenting with the highest mean number of affected teeth, and CP patients presenting with the lowest mean number of affected teeth (Fig. 2). The Kruskal-Wallis H test indicated a nonsignificant difference between these groups [χ2(4) = 4; p = 0.406].

Table 3 summarises the teeth most commonly affected by each dental anomaly type and their relative locations to the cleft site. The vast majority of dental anomalies affected teeth within or close to the region of the cleft.

**Table 3:** Teeth most commonly affected by each dental anomaly, and their location relative to the cleft site

Dental anomaly	Teeth commonly affected by anomaly (%)	Location of aforementioned teeth commonly affected by anomaly (%)
Tooth agenesis	Upper lateral incisors (71%)	Same side as cleft (91%)
Ectopic eruption/impaction	Upper canines (31%)	Same side as cleft (56%)
Enamel HM/P	Upper central and lateral incisors (75%)	N/A
Shape and/or size abnormality	Upper lateral incisors (90%)	Same side as cleft (74%)
Supernumerary	Upper lateral incisors area (48%)	Same side as cleft (100%)

**Fig. 3:**
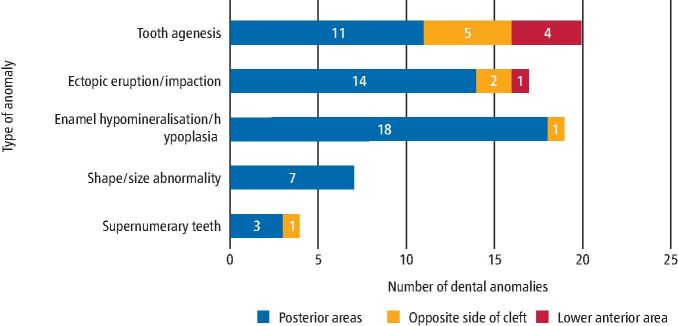
Dental anomalies outside the cleft region, and their relative locations to the cleft site

A total of 46 patients (31.9%) had one or more dental anomalies outside the cleft site region (Fig. 3), the majority of which were located in posterior quadrants of the mouth. With reference to tooth agenesis, 100% (n = 11) of these anomalies within posterior areas affected premolars. Ectopic eruption/impaction anomalies within posterior areas included: infra-occluding primary molars (n = 6; 42.8%); impacted premolars and first permanent molars; and transposed premolars. Anomalies of enamel HM/P within posterior areas mainly affected first permanent molars (n = 12; 66.7%), followed by premolars and primary molars.

## Discussion

When compared with unaffected individuals, patients with CLP have been found to have a higher prevalence of dental anomalies,^6^^,^^8^^,^^9^ with anomalies varying according to the CLP category and higher frequencies identified as the severity of the cleft increases.^4^^,^^6^^,^^10^ This is consistent with the findings of this study, with a greater proportion of BCLP and UCLP cleft types presenting with 3-5 different dental anomaly types.

Additionally, this study showed that the second most common dental anomaly was tooth agenesis, affecting 41.6% (n = 60) of all patients, and was most prevalent in BCLP and UCLP cleft types, which was significant. Although the aetiology of dental anomalies is still not quite clear, it has been demonstrated in the last decade that genetic factors play a major role in dental agenesis, with mutations in MSX1 and PAX9 genes having been associated with non-syndromic tooth agenesis in humans within and outside the cleft area,^10^^,^^11^^,^^12^ causing the combined development of orofacial clefts and hypodontia.^13^^,^^14^

Dental agenesis was most common in individuals with a complete CLP compared to those with CL, CLA and CP only. This finding corroborates previous studies, where the number of missing teeth was higher as cleft severity increased.^14^ Given that in these cases the cleft region has a bone defect, it is not surprising that teeth close to the cleft are commonly malformed or missing.^6^^,^^15^ This study, however, did not take into account poorly malformed teeth, which may have been extracted from the cleft site during bone grafting, either due to poor long-term prognosis or to allow the surgeon access to the cleft site.

The most common dental anomaly within this study was enamel HM/P, affecting 45.8% (n = 66) of all patients; these consisted of approximately 50% of all CLP types, but only 25% of CP-only patients. Additionally, 75% of these enamel defects were located on upper central and lateral incisor teeth. Some studies have attributed the high prevalence rate of this anomaly, especially in the upper incisor area, to the surgical repair of the lip and palate.^16^ The reason for this is that the primary lip and secondary palate repair surgeries, which normally take place around 3-6 months and 9-12 months, respectively, coincide with the calcification of maxillary permanent incisors.^11^^,^^16^

Supernumerary teeth anomalies illustrated an intriguing pattern. Patients with a malformation of the primary palate solely (CLA) or CL only appeared to be more often affected by supernumerary teeth than patients with cleft of the lip and palate. This was a significant finding in our study and reflects the literature, where the frequency of supernumerary teeth increased with the severity of cleft decreasing.^10^^,^^17^ This could be attributed to the extent of the cleft and its effect on the epithelium forming the dental germs. If a smaller extension of the cleft stops the epithelium from uniting, a supernumerary tooth is formed, while a larger cleft may cause microdontia, and an even greater lack of epithelium may cause tooth agenesis.^13^^,^^18^

Therefore, although the frequency of dental anomalies increases as the severity of the cleft increases within the bony tissue harbouring the tooth buds, one should not assume that CL-only patients are unaffected by dental anomalies. A large proportion of CL-only patients (n = 15; 88%) within the study had dental anomalies, despite their cleft only involving the lip. Additionally, CP-only patients, where the cleft is located within the palate rather than the alveolar bone retaining the tooth buds, though showing a lower prevalence comparable to previous research, were shown to be affected by dental anomalies in almost half of the cases reviewed.

Within the study, the vast majority of dental anomalies affected teeth on the same side as the cleft, in regions within or close to the cleft region. This reinforces the literature, as studies have shown that dental anomalies occur more frequently on the cleft side affecting both permanent and deciduous teeth, with the maxillary lateral incisors being the most disposed to dental anomalies within the cleft region^4^^,^^19^ due to their positioning in relation to the cleft.

With regards to dental anomalies outside the cleft site (31.9%), the majority of these were located in posterior quadrants of the mouth. This isn't an area which is normally reported on within the literature. The findings within this study indicated that despite being outside the cleft region, tooth agenesis and enamel HM/P still remained the most common dental anomalies. We also know that MSX1 and PAX9 genes are associated with tooth agenesis both within and outside the cleft region, and in many instances, with preferential premolar agenesis.^14^^,^^20^ It has also been suggested that dental anomalies affecting maxillary lateral incisors on the opposite side of the cleft could indicate an unsuccessful bilateral cleft.^14^ The distribution of these anomalies may therefore suggest a common genetic and environmental background of both the cleft and the concomitant anomalies.^16^

This study, however, did not consider the impact sex or race may have on the incidence of dental anomalies within our patient cohort. Prospective research may therefore be of benefit in investigating whether these factors contribute to both the incidence and distribution of these.

### Implications for dentists working in primary care

Dental anomalies may complicate the dental management of these patients and therefore good early experiences are pivotal in creating positive attitudes towards dentistry throughout childhood and adulthood.

Cleft patients will frequently be seen by a cleft multi-disciplinary team (MDT), of which there are 17 across the UK. These consist of: a cleft surgeon; a specialist in paediatric dentistry; a consultant orthodontist; a consultant in restorative dentistry; a specialist/consultant in paediatric dentistry; a specialist speech and language therapist; a clinical psychologist; and a consultant ear, nose and throat surgeon, among others.^21^^,^^22^ It is also important to note that access to cleft services is available throughout a patient's life and they can therefore return for further treatment/advice at any age.

From a dental perspective, children with a cleft are initially seen by the specialist/consultant in paediatric dentistry within their local cleft team at around 12 months of age to offer dental health advice, preventive treatment and a referral to other services if required. Routine dental care is ideally undertaken and provided by the child's local general dental practitioner. As the patient grows older and attends further cleft MDT clinics, which include treatment planning and long-term management with orthodontic and restorative input, general dental practitioners are paramount in the shared care of these patients.^23^^,^^24^

Early identification of dental anomalies is essential. Although this study explored the most commonplace dental anomalies found in cleft patients, it is worth noting that the literature also mentions other dental anomalies which may present themselves in this population. These include: *dens invaginatus, dens evaginatus,* pulp stones and taurodontism.^8^ In light of the above, teeth with *dens invaginatus* in particular should be fissure sealed as soon as they are identified following eruption, in order to reduce the risk of devitalisation.

In view of the high prevalence of tooth agenesis, ectopic impactions, and lateral incisors of poor morphology, maintaining teeth and spacing is often a priority within the cleft population. Patients are likely to require orthodontic treatment as they progress through the cleft pathway. Enhanced prevention following the *Delivering better oral health toolkit*^25^ is therefore critical in ensuring patients maintain good oral health. Additionally, the high prevalence of enamel HM/P makes these teeth more susceptible to dental caries. It is known that cleft-affected individuals have a higher caries prevalence compared their non-cleft counterparts.^23^^,^^24^^,^^26^^,^^27^

As treatment plans are formulated through cleft MDTs, certain teeth may require treatment within primary dental care. For instance, shape/size anomalies may require restoration or build-up to camouflage, while certain teeth within the cleft site may require extraction if they are deemed to be of poor long-term prognosis. Additionally, cleft patients planned for alveolar bone grafting between the ages of 9-11 years old, will need to be 'dentally fit' before the procedure, which may involve the provision of dental restorations or extractions.^23^^,^^24^ When undertaking treatment, considerations must be made of the patient's medical history; this will be explored further within Part 2.^7^

## Conclusion

This article gives a greater understanding of the multitude of dental anomalies that can affect CLP patients, an understanding crucial to their successful management and alleviation of aesthetic and functional concerns.

The presence of dental anomalies is strongly correlated to the presence of clefts (including CL-only), with certain anomalies being more prevalent in certain cleft types; the most common being enamel HM/P and tooth agenesis, which is consistent with previous studies. The severity of the dental anomalies seems to be directly related to the severity of the cleft within the bony tissue harbouring the tooth buds, with the vast majority of dental anomalies affecting teeth within or close to the region of the cleft. Although this study suggests that tooth agenesis and enamel HM/P remained the most common dental anomalies outside the cleft region, the literature regarding anomalies outside the cleft region is limited.

The identification of these dental anomalies is important when treatment-planning cleft patients in a multidisciplinary setting, highlighting the importance of specialist paediatric dental input at cleft MDT clinics to identify these anomalies early and manage them long-term. Intensive preventive regimes and shared care with dentists working in primary care is paramount and crucial, not only to the holistic care of these patients, but also their long-term outcomes.
